# *Echinococcus multilocularis* in Northern Hungary

**DOI:** 10.3201/eid1007.031027

**Published:** 2004-07

**Authors:** Tamás Sréter, Zoltán Széll, Zsuzsanna Sréter-Lancz, István Varga

**Affiliations:** * Central Veterinary Institute, Budapest, Hungary;; † National Food Investigation Institute, Budapest, Hungary;; ‡ Szent István University, Budapest, Hungary

**Keywords:** letter, *Echinococcus multilocularis*, *Vulpes vulpes*, red fox, *Arvicola terrestris*, water vole, water-spreading, Hungary, Europe

**To the Editor:** *Echinococcus multilocularis* infection is one of the most dangerous zoonoses in the Northern Hemisphere and causes more human death than rabies in Europe. Recent data indicate that *E. multilocularis* infection is spreading geographically and is being transmitted at an increasing rate in Europe (Figure). Since 1995, the parasite has been found in Poland, the Czech Republic, the Slovak Republic, Belarus, Hungary, and Romania; infections in humans have been increasing in frequency in central eastern Europe since the late 1990s (1–4). Since the 1990s, similar infection trends in foxes and humans have been observed in central western European countries, including eastern Austria, northern Germany, Denmark, the Netherlands, Luxembourg, and Belgium (1,2,5). Despite the increasing prevalence of *E. multilocularis* infection in foxes, the number of human cases did not vary in the historically known area (eastern France, Switzerland, southern Germany, and western Austria) during the 1990s (1), probably because of increased public awareness and control measures. In our previous study (2), the parasite was detected only in foxes in the Hungarian-Slovak border area in northeastern Hungary. In the current study, we found the parasite distributed along the watershed area of the River Danube in northern Hungary.

**Figure:**
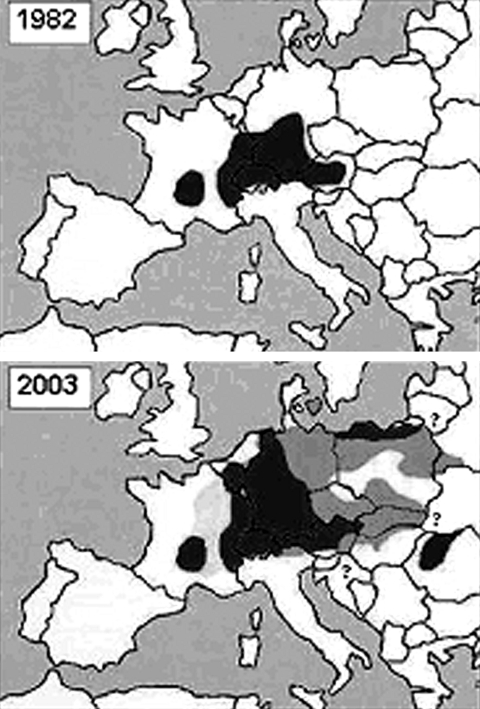
Distribution of *Echinococcus multilocularis* in Europe (1,2,4, this study). Black areas: Infection was reported in men, foxes and or rodents. Dark gray areas: Infection was described only in foxes and or rodents. Light gray areas: Only human cases were noted. White areas: *E. multilocularis* free territories. Question marks: The presence or appearance of the parasite is projected. Note: The prevalence of infection in foxes is similar in the majority of the affected countries.

In the second half of 2003, carcasses of red foxes were sent to the Central Veterinary Institute in Budapest for examination in connection with the rabies immunization and control program in seven counties (Vas, Györ-Sopron, Komárom-Esztergom, Pest, Nógrád, Heves, and Borsod-Abaúj-Zemplén) that bordered or were near the border of Austria and the Slovak Republic. These foxes were included in the current study. Methods of transporting and storing the carcasses, examining the intestinal tracts, and identifying parasites have been described previously (2).

Of 150 foxes examined, 19 animals from four counties (Györ-Sopron, Komárom-Esztergom, Pest, and Nógrád) harbored 2, 3, 4, 6, 7, 14, 22, 31, 51, 54, 114, 130, 200, 250, 300, 400, 800, and 1,300, and 5,500 mature worms of *Echinococcus*. On the basis of the most important morphometric guidelines and the results of the species-specific polymerase chain reaction (PCR) assay, the parasites were identified as *E. multilocularis*. The examined foxes were found from 5 to 70 km from the known endemic areas of the Slovak Republic, Austria, and Hungary, and from 5 to 50 km from the northern border of the country. On the basis of this information and the previous study (2), the overall prevalence rate of infection was 16% (24/156) in the five northern counties of Hungary (Györ-Sopron 30%, Nógrád 26%, Komárom-Esztergom 7%, Pest 6%, and Borsod -Abaúj-Zemplén 5%). This prevalence rate is similar to those observed in Belgium, Poland, and the Slovak Republic in recent years. In these countries, a total of 30 human cases have been reported since 1995 (3–6).

In Germany, infected foxes were more frequently found near water (7), which indicates a water-related natural cycle of the parasite. The spatial aggregation analysis of the parasite in intermediate hosts demonstrated that areas with humid conditions are at high risk for human exposure (8). In Europe, the most important water-related intermediate host of *E. multilocularis* is the water vole (*Arvicola terrestris*) (9). The prevalence of *E. multilocularis* in water voles can be as high as 39% in disease-endemic areas (10). Areas with high water-vole densities yielded a 10-fold higher risk for alveolar echinococcosis in humans compared to areas with low densities (10). These data indicate that water voles may play an important role in the epidemiology of *E. multilocularis*. All infected foxes included in this and the previous study (2) were found near permanent natural waters, i.e., in those areas where water vole populations exist, such as Lake Fertö, the River Danube, the River Ipoly, the River Rába, and several streams connected to the watershed area of the River Danube. *E. multilocularis* might have spread in the northern part of Hungary along the watershed area of the River Danube, coming from the known endemic areas of Austria and the Slovak Republic. Similar spreading of the parasite along waterways was also observed in the Slovak Republic (11).

In the historically known *E. multilocularis*–endemic mountain areas, both fossorial and aquatic water voles exist (12). The density of these populations can be 10-fold greater than that of aquatic populations in other European countries (12). On the basis of the long incubation period of the parasite in humans (5–15 years) and the dates of the first human cases reported outside the historically known area (Figure), foxes might have reached the population density needed (13) to maintain the parasite cycle in low water-vole density areas in Europe from the 1980s (Figure). Although the parasite crossed the border of several countries that surrounded the known area, further spreading was not observed in those countries where *A. terrestris* is an endangered species (the Netherlands, northern Italy) or where water voles are absent from the fauna (western and southern France) (12).

The River Danube and several small streams crossing Budapest, the capital of Hungary with a population of 2 million, create ideal circumstances for urbanization of the life cycle of a parasite that involves water voles and red foxes. Urbanization of the life cycle of *E. multilocularis* was recently observed in Prague, the capital of the Czech Republic with a population of 1 million and similar hydrographic features (14); therefore, occurrences of this zoonosis should be continuously monitored in Budapest. Further studies are necessary to monitor the possible spread of the parasite in other regions that are thought to be currently free of the infection.

The regulatory, veterinary, and public health authorities of the European Union mobilized considerable financial and human resources to control rabies and paid less attention to alveolar echinococcosis in the 1990s, although incidence data indicate that alveolar echinococcosis is increasing and became an emerging infectious disease in Europe. In the Directive 2003/99/EC of the European Parliament and of the Council repealing Council Directive 92/117/EEC, echinococcosis has been added to the list of zoonoses to be monitored in the European Union countries. Effective methods to control *E. multilocularis* are unavailable; however, the zoonosis should be monitored and evaluated, and development of control programs should be intensified.
